# *Serpentinimonas* gen. nov., *Serpentinimonas raichei* sp. nov., *Serpentinimonas barnesii* sp. nov. and *Serpentinimonas maccroryi* sp. nov., hyperalkaliphilic and facultative autotrophic bacteria isolated from terrestrial serpentinizing springs

**DOI:** 10.1099/ijsem.0.004945

**Published:** 2021-08-11

**Authors:** Lina J. Bird, J. Gijs Kuenen, Magdalena R. Osburn, Naotaka Tomioka, Shun’ichi Ishii, Casey Barr, Kenneth H. Nealson, Shino Suzuki

**Affiliations:** ^1^​ Center for Bio/Molecular Science and Engineering Naval Research Lab, 4555 Overlook Ave S.W., Washington DC 20375, USA; ^2^​ Department of Earth Sciences, University of Southern California, 35 W. 37th St. SHS 560, Los Angeles, California 90089, USA; ^3^​ Department of Biotechnology, Delft University of Technology, van der Maasweg 9, 2629HZ, Delft, Netherlands; ^4^​ Department of Earth and Planetary Sciences, Weinberg College of Arts & Sciences. Northwestern University Evanston, Evanston, USA; ^5^​ Kochi Institute for Core Sample Research, Japan Agency for Marine-Earth Science and Technology (JAMSTEC), Monobe B200, Nankoku, Kochi 783-8502, Japan; ^6^​ Institute for Extra-cutting-edge Science and Technology Avant-garde Research (X-star), JAMSTEC, Natsushima 2-15, Yokosuka, Kanagawa 237-0061, Japan; ^7^​ Institute of Space and Astronautical Science (ISAS), Japan Aerospace Exploration Agency (JAXA), 3-1-1 Yoshinodai, Chuo-ku, Sagamihara, Kanagawa 252-5210, Japan

**Keywords:** alkaliphile, autotrophic growth, Serpentinization, *Serpentinomonas/Serpentinimonas*

## Abstract

Three highly alkaliphilic bacterial strains designated as A1^T^, H1^T^ and B1^T^ were isolated from two highly alkaline springs at The Cedars, a terrestrial serpentinizing site. Cells from all strains were motile, Gram-negative and rod-shaped. Strains A1^T^, H1^T^ and B1^T^ were mesophilic (optimum, 30 °C), highly alkaliphilic (optimum, pH 11) and facultatively autotrophic. Major cellular fatty acids were saturated and monounsaturated hexadecenoic and octadecanoic acids. The genome size of strains A1^T^, H1^T^ and B1^T^ was 2 574 013, 2 475 906 and 2 623 236 bp, and the G+C content was 66.0, 66.2 and 66.1 mol%, respectively. Analysis of the 16S rRNA genes showed the highest similarity to the genera *Malikia* (95.1–96.4 %), *Macromonas* (93.0–93.6 %) and *Hydrogenophaga* (93.0–96.6 %) in the family *Comamonadaceae *. Phylogenetic analysis based on 16S rRNA gene and phylogenomic analysis based on core gene sequences revealed that the isolated strains diverged from the related species, forming a distinct branch. Average amino acid identity values of strains A1^T^, H1^T^ and B1^T^ against the genomes of related members in this family were below 67 %, which is below the suggested threshold for genera boundaries. Average nucleotide identity by blast values and digital DNA–DNA hybridization among the three strains were below 92.0 and 46.6 % respectively, which are below the suggested thresholds for species boundaries. Based on phylogenetic, genomic and phenotypic characterization, we propose *Serpentinimonas* gen. nov., *Serpentinimonas raichei* sp. nov. (type strain A1^T^=NBRC 111848^T^=DSM 103917^T^), *Serpentinimonas barnesii* sp. nov. (type strain H1^T^= NBRC 111849^T^=DSM 103920^T^) and *Serpentinimonas maccroryi* sp. nov. (type strain B1^T^=NBRC 111850^T^=DSM 103919^T^) belonging to the family *Comamonadaceae *. We have designated *Serpentinimonas raichei* the type species for the genus because it is the dominant species in The Cedars springs.

The family *Comamonadaceae *, which belongs to the class *Betaproteobacteria *, was first described by Willems *et al*. [1] and now contains at least 50 genera. Most members of this family were isolated from soil, freshwater, activated sludge, hot springs and pond water [2–8]. These genera harbour a phenotypic diversity that includes aerobic organotrophs [2], anaerobic denitrifiers [5, 9], Fe^3+^-reducing bacteria [10], hydrogen oxidizers [11], and phosphate-accumulating and -removing bacteria [6, 12], and cyclohexane-degrading bacteria [13]. In this study, we characterize three hyper-alkaliphilic strains (A1^T^, H1^T^ and B1^T^) isolated from an active terrestrial serpentinization site that represents an unusual microbial habitat, as the fluids are highly alkaline, enriched in calcium, low in sodium and have abundant dissolved hydrogen gas [14–16]. Given that relatives of strains A1^T^, H1^T^ and B1^T^ were dominant in various terrestrial active serpentinizing sites, these strains are likely relevant to the geochemistry of terrestrial serpentinization sites. We expect future studies on the ecology, physiology, biochemistry and molecular genetics of these organisms to contribute to a better understanding of life under these extremely alkaline conditions.

The samples were collected from Barnes Spring 1 (BS1) and Barnes Spring 5 (BS5; elevation 282 m, N: 38° 37.282′, W: 123° 07.987′) located at The Cedars serpentinization site in northern California as described previously [17, 18]. Briefly, samples of pre-autoclaved glass beads (0.11 mm diameter ballotini beads) that were incubated *in situ* in the BS1 pool at E_h_ of around −250 mV for 1 week and then collected for the isolation of strains A1^T^ and B1^T^. Strain H1^T^ was isolated from a sample of pool BS5 water. The samples were inoculated in sterile Cedars standard medium (CSM) 1 containing 0.05 mm Na_2_SO_4_, 0.378 mm NH_4_Cl, 0.05 mm MgCl_2_, 0.06 mm K_2_HPO_4_, 10 mm CABS or Na_2_CO_3_/NaHCO_3_, 2 mm CaCO_3_ (as suspension), 4 mm sodium acetate, 10 ml l^−1^ of ATCC trace mineral supplement and 10 ml l^−1^ of ATCC vitamin solution. The pH was adjusted to pH 10.5–11.2 using NaOH. Gas phase of the media in stoppered serum vials was replaced with a mixture of O_2_/H_2_/N_2_/Ar (2.6 : 50 : 9.8 : 37.6 by volume at 1 atm). After incubation for 2 weeks at 16–18 °C, the sample suspension was streaked on CSM1 agar plates containing CSM1 with 2 % prewashed noble (Difco) or Korean (Daishin) agar at pH 10.5, and incubated under the same gas mixture. Streaked plates were incubated at 18 °C for 2 weeks.

Creamy/opaque colonies of strains A1^T^, B1^T^ and H1^T^ were purified after repeatedly culturing on the CSM1 agar plates at 18 °C for 2 weeks. Colonies of strains A1^T^ and B1^T^ were suspended in glycerol diluted by CSM1 (20 % v/v) and stored at −80 °C. Strain H1^T^ purified on the plate was incubated at 16 °C in liquid CSM1 and concentrated by centrifugation and then stored in glycerol diluted by CSM1 (20 % v/v) at −80 °C.

DNA extraction from the strains and determination of genome sequences have been described previously [19]. The complete genomes of two strains, A1^T^ and B1^T^, and the draft genome of strain H1^T^ have been reported previously [19]. The 16S rRNA gene nucleotide sequences of the strains were retrieved from the genome sequences and aligned with 24 reference sequences from public databases using the program muscle [20]. Strains A1^T^, H1^T^ and B1^T^ were closely related to the type species of three genera including *Malikia granosa* P1^T^ (95.3, 96.4, 96.3 % 16S rRNA gene sequence identities, respectively), *Macromonas bipunctata* DSM 12705^T^ (93.0, 93.5, 93.6 %) and *Hydrogenophaga flava* DSM 619^T^ (95.3, 96.3, 96.6 %). Among the three strains, 16S rRNA gene sequence identity between strains A1^T^ and H1^T^ was 98.9 %, between strains A1^T^ and B1^T^ it was 97.6%, and between strains B1^T^ and H1^T^ it was 98.7 %.

A phylogenetic tree was created by using the maximum-likelihood method with RaxML [21]. The robustness of furcated branches was supported by bootstrap values (1000 replicates) (Fig. 1). The topology was further confirmed by neighbour-joining. Phylogenetic analysis based on 16S rRNA genes revealed that strains A1^T^, H1^T^ and B1^T^ belong to the family *Comamonadaceae* in the order *Burkholderiales *. The three isolates formed a distinct branch separate from other members of *Comamonadaceae* with the bootstrap value of 100 (Fig. 1). The closest relatives of the three isolates were the genera *Macromonas* [4], *Malikia* [6] and *Hydrogenophaga* [11].

**Fig. 1.**
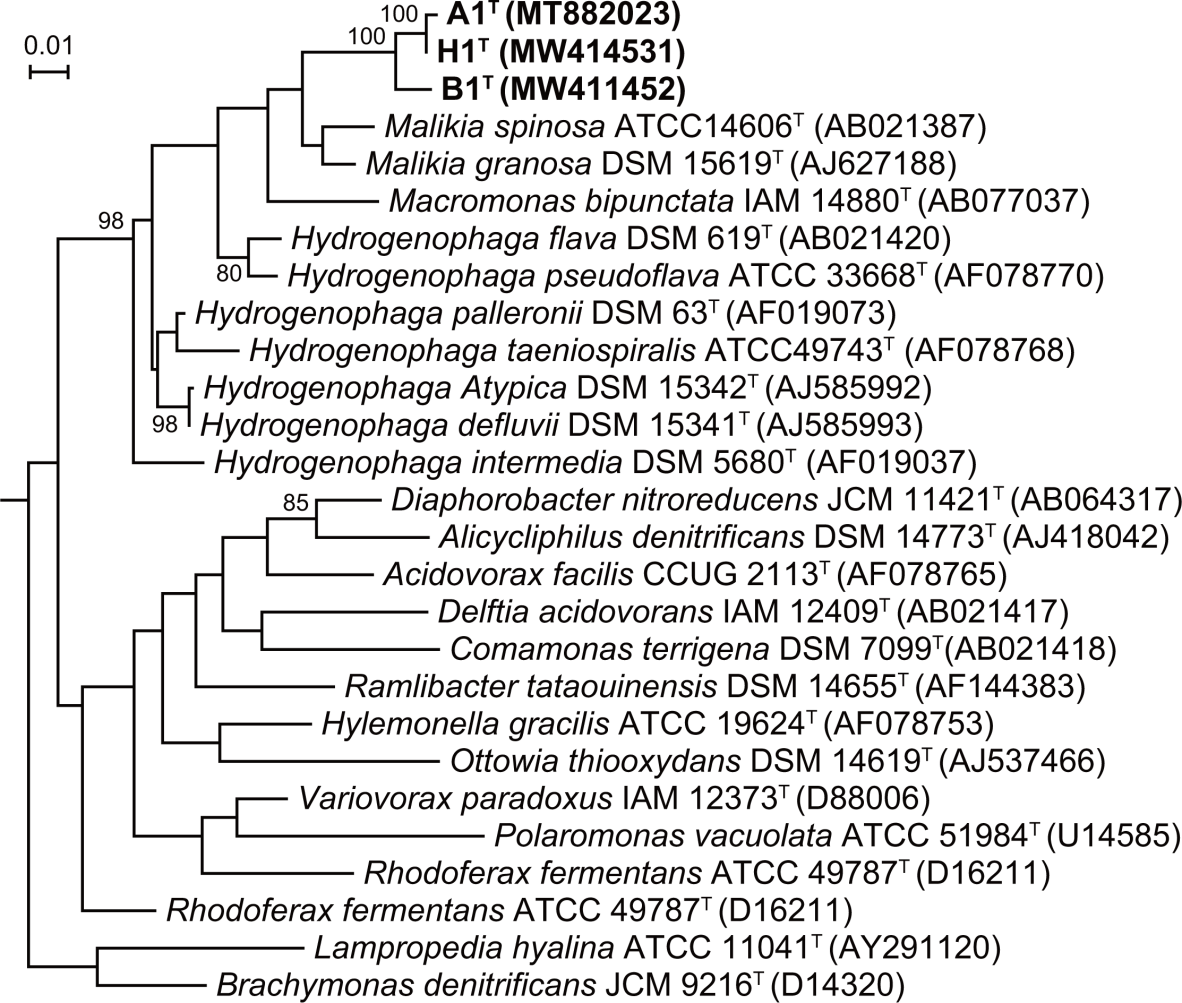
Phylogenetic relationship derived from 16S rRNA gene sequences between strains A1^T^, B1^T^ and H1^T^ and other related taxa of the family *Comamonadaceae *. The tree was reconstructed using the maximum-likelihood method based on 16S rRNA gene sequences. Bootstrap values greater than 80 % are shown at branch points. Bar, 0.02 substitutions per nucleotide position.

The genome sizes of strains A1^T^, H1^T^ and B1^T^ were 2 574 013, 2 475 906 and 2 623 236 bp, and the G+C contents were 66.0, 66.2 and 66.1 mol%, respectively. CheckM analysis showed that the genome completeness of the strains was over 99 % [22]. Thirty conserved marker genes were extracted from the 14 genomes in the family *Comamonadaceae* and *Burkholderia cepacia* in the family *Burkholderiaceae *, and a concatenated alignment (5823–6689 amino acids) was generated in the CheckM platform [23]. A maximum-likelihood phylogenomic tree based on the concatenated alignment was generated by using mega X [24] and the JTT matrix-based model with 100 resamples. This phylogenomic tree indicated that strains A1^T^, H1^T^ and B1^T^ are deeply branching among those of closely related genera, as seen in the tree based on 16S rRNA genes (Fig. 2).

**Fig. 2.**
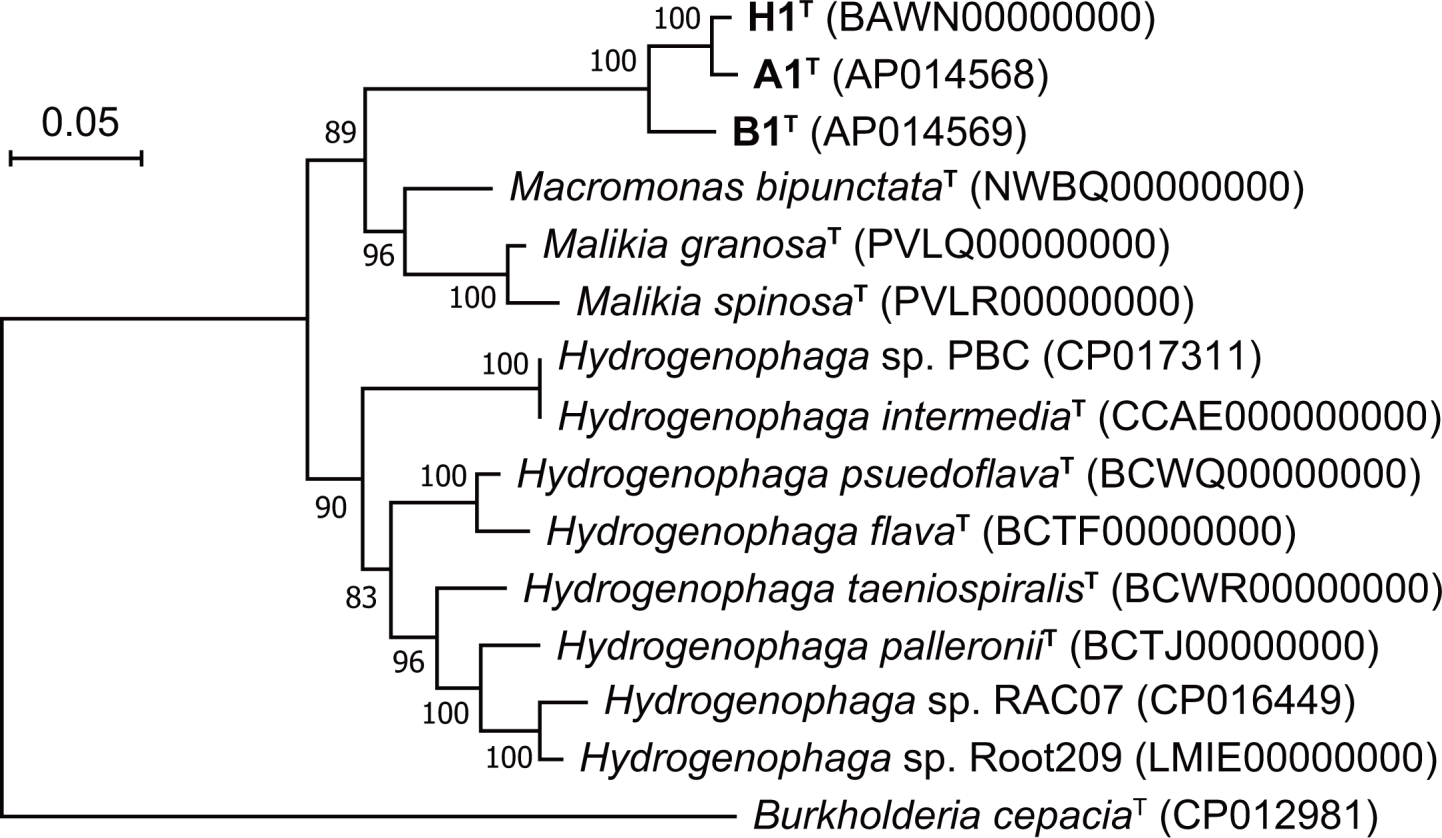
Phylogenomic relationship based on concatenated alignment of amino acid sequences between strains A1^T^, B1^T^ and H1^T^ and other related taxa of the family *Comamonadaceae *. The tree was reconstructed using the maximum-likelihood method based on concatenated alignment of amino acid sequences of 30 conserved marker genes coded in the genomes. Bootstrap values are shown at branch points.

Amino acid identity (AAI) values of the strains against the genomes of closely related genera in the family *Comamonadaceae* were obtained using the Kostas lab AAI calculator web server (http://enve-omics.ce.gatech.edu/aai/) [23]. In the past, AAI values between 60–80 % were taken as thresholds for distinguishing genera [25, 26]. However, recent studies of new genus descriptions in the family *Comamonadaceae* and other phylum proposed that the threshold for genera boundaries should be 70 % for AAI [8, 27]. The three isolated strains showed the highest AAI values to genus *Macromonas *, with a value of 67 %. The next highest AAI values were 65–66 % with the genus *Malikia *, while AAI values with the genus *Hydrogenophaga* were 64–65 % (Table 1). All the AAI values were lower than the proposed genus boundary threshold [27], making the strains A1^T^, H1^T^ and B1^T^ distinct from previously described genera.

**Table 1.** AAI, ANIb and dDDH results for the three strains with genera *Macromonas *, *Malikia* and *Hydrogenophaga
* Top, AAI values from the genome-based distance matrix calculator. Bold letters are above genus cut-off values (70 %). Bottom, ANIb values from JSpeciesWS and dDDH values derived from the Genome-to-Genome Distance Calculator (in parentheses). Bold letters are above species cut-off values (70 % for dDDH and 96 % for ANIb).

	A1^T^	H1^T^	B1^T^	* Macromonas bipunctata *	* Malikia granosa *	* Malikia spinosa *	* Hydrogenophaga flava *	*Hydrogenophaga psuedoflava*	* Hydrogenophaga taeniospiralis *	*Hydrogenophaga*sp. PBC	* Hydrogenophaga intermedia *	*Hydrogenophaga*sp. RAC07	*Hydrogenophaga*sp. Root209	* Hydrogenophaga palleronii *	Total length (bp)	G+C content (mol%)	No. of sccafold	Refseq accession no.
**A1^T^**	*	**93**	**85**	67	65	65	65	65	65	64	64	65	64	65	2 574 013	66.0	1	GCF_000828895.1
**H1^T^**	92.0 (46.6)	*	**85**	67	66	66	65	65	65	65	65	65	65	65	2 475 906	66.2	93	GCF_000696225.1
**B1^T^**	85.3 (29.6)	85.0 (28.8)	*	67	65	65	64	65	65	64	64	64	64	64	2 623 236	66.1	2	GCF_000828915.1
* Macromonas bipunctata *	75.1 (20.5)	75.3 (20.6)	75.3 (20.5)	*	**71**	**71**	66	67	66	65	65	66	66	66	2 699 505	63.8	115	GCF_002837135.1
* Malikia granosa *	74.4 (20.3)	74.6 (20.5)	74.7 (20.9)	77.7 (22.4)	*	**90**	66	66	66	64	64	65	65	65	3 832 968	66.8	162	GCF_002980595.1
* Malikia spinosa *	74.4 (20.2)	74.4 (20.2)	74.6 (20.8)	77.5 (22.4)	89.8 (40.3)	*	65	66	65	63	64	65	64	65	3 778 566	65.6	103	GCF_002980625.1
* Hydrogenophaga flava *	73.2 (19.8)	73.2 (19.9)	73.4 (19.9)	75.6 (21.3)	75.1 (21.3)	74.8 (21.1)	*	**94**	**77**	69	69	**74**	**73**	**72**	5 161 958	67.1	82	GCF_001571145.1
*Hydrogenophaga psuedoflava*	73.3 (19.6)	73.5(19.8)	73.5(20.2)	75.7(21.2)	75.6(21.2)	75.2(21.1)	92.5(50.7)	*	**78**	**70**	**70**	**74**	**74**	**73**	4 505 692	67.3	43	GCF_001592285.1
* Hydrogenophaga taeniospiralis *	73.5 (19.9)	73.6(20.2)	73.6(20.4)	75.9(21.8)	75.1(21.8)	74.9(21.5)	80.4(24.5)	80.2(24.3)	*	**70**	**70**	**76**	**76**	**74**	5 275 331	66.7	53	GCF_001592305.1
*Hydrogenophaga*sp. PBC	73.5 (20.1)	73.6 (20.3)	74.0 (20.8)	75.5 (20.6)	74.6 (20.9)	74.2 (20.7)	76.5 (21.4)	76.7 (21.9)	77.1 (22.2)	*	**100**	**74**	**73**	**74**	5 144 529	68.4	1	GCF_000263795.2
* Hydrogenophaga intermedia *	73.4 (20.1)	73.5 (20.2)	73.8 (20.5)	75.4 (20.6)	74.3 (20.8)	74.3 (20.8)	76.6 (21.4)	76.8 (21.8)	77.2 (22.3)	**99.7**(99.2)	*	**74**	**73**	**73**	5 288 135	68.4	124	GCF_000723405.1
*Hydrogenophaga*sp. RAC07	73.0 (19.8)	73.2 (20.1)	73.2 (20.3)	75.4 (20.8)	74.3 (20.6)	74.2 (20.9)	77.5 (21.8)	77.5 (21.9)	79.5 (24.1)	77.6 (21.5)	77.6 (21.5)	*	**89**	**79**	4 674 680	65.5	1	GCF_001713375.1
*Hydrogenophaga*sp. Root209	72.8 (19.4)	73.1 (19.6)	73.0 (19.9)	75.3 (20.7)	74.4 (21.4)	73.9 (20.6)	77.1 (21.8)	77.2 (22.0)	79.2 (24.1)	77.1 (21.8)	77.0 (21.7)	86.5 (34.2)	*	**79**	5 307 743	65.1	34	GCF_001428625.1
* Hydrogenophaga palleronii *	73.5 (20.0)	73.7 (19.9)	73.7 (20.6)	75.9 (21.2)	74.8 (21.2)	74.6 (21.0)	77.6 (22.5)	77.9 (22.7)	79.4 (24.1)	78.2 (22.6)	78.1 (22.6)	80.7 (25.3)	80.5 (25.3)	*	4 841 746	66.8	110	GCF_001571225.1

Average nucleotide identity by blast (ANIb) values and digital DNA–DNA hybridization (dDDH) were calculated by using JSpecies [28] and the DSMZ Genome-to-Genome Distance Calculator platform [29], respectively. The ANIb and dDDH values among the three isolated strains were 85.0–92.0 % and 28.8–46.6 %, respectively (Table 1), which are lower than the delineation of species boundaries (ANIb <95 % and dDDH <70 %) [30].

The purified strains were initially grown and tested on CSM2, which contains 0.1 mm Na_2_SO_4_, 0.755 mm NH_4_Cl, 0.1 mm MgCl_2_, 0.23 mm K_2_HPO_4_, 20 mm CaCO_3_ (as suspension), 15 mm CAPS buffer (pH 11), 10 ml l^−1^ ATCC trace mineral supplement and 10 ml l^−1^ ATCC vitamin solution. The portion for the liquid and gas phase was approximately 35 : 65. The gas composition was H_2_/N_2_/air 35 : 35 : 30, the temperature for the cultivations is at 30 °C. Further optimization led to CSM3, which contained 0.2 mm Na_2_SO_4_, 1.5 mm NH_4_Cl, 0.199 mm MgCl_2_, 0.23 mm K_2_HPO_4_, 5 mm CaCl_2_, 10 ml l^−1^ ATCC vitamin solution, 10 ml l^−1^ ATCC mineral solution and 15 mm CAPS buffer (pH 11) and the gas composition was H_2_/N_2_/air 35 : 35 : 30 for autotrophic growth and N_2_/air 75 : 30 for heterotrophic growth. Calcium, phosphate, vitamins and minerals were added as separate filter-sterilized solutions after autoclaving the basal salt solution. For autotrophic growth, the CaCl_2_ in CSM3 was replaced with 20 mm CaCO_3_, while 3 mm acetate was used for routine heterotrophic growth. Substrate utilization were tested in CMS2. pH range and optimum pH were tested in both CSM2 and CSM3. Growth rate determination, antibiotic sensitivity, catalase assay, Gram staining, light microscopy, TEM and SEM, cytochrome analysis, and lipid analysis were performed in CSM3. When testing utilization of organic substrates, a substrate was added to the CSM2 and CSM3. For testing the anaerobic growth, medium was thoroughly flushed with N_2_. The results of substrate utilization are shown in Table 2. All three strains were able to grow autotrophically using hydrogen, calcium carbonate and oxygen or heterotrophically on a variety of electron donors, although strain A1^T^ grew poorly in the absence of hydrogen. None of the strains could utilize sulphate, iron (III) hydroxide, or iron (II/III) oxide as electron acceptors. All strains grew best aerobically on sub-atmospheric levels of oxygen (1–4 % v/v). During mixotrophic growth on hydrogen and acetate, the minimum doubling time was 10 h for strain A1^T^, 12.5 h for strain H1^T^ and 8.5 h for strain B1^T^, respectively.

**Table 2.** Differentiating characteristics of strains A1^T^, H1^T^ and B1^T^ from closely related type strains in the family *Comamonadaceae
* Strains: 1, A1^T^ (data from this study and [19]); 2, H1^T^ (data from this study and [19]); 3, B1^T^ (data from this study and [19]); 4, *Malikia granosa* P1^T^ [6]; 5, *Hydrogenophaga flava* DSM 619^T^ [11, 40]; 6, *Hydrogenophaga palleronii* DSM 63^T^ [11, 40, 41]; 7, *Macromonas bipunctata* DSM 12705^T^ [6]. +, Positive; −, negative; w, weak positive; nt, not tested or not available in the literature

Characteristics	1	2	3	4	5	6	7
Isolation source	Serpentinized water	Serpentinized water	Serpentinized water	Activated sludge	Mud	Soil and water	Slime
Colony colour	Opaque cream	Opaque cream	Opaque cream	Cream white	nd	Pale yellow	nd
Temperature for growth (°C)	18–37	18–37	18–37	<40	<42	<42	nd
Optimum temperature for growth (°C)	26–30	26–30	26–30	35	30	30	28
pH range for growth	10.0–11.5	9.0–12.0	9.5–12.5	nd	nd	nd	nd
Optimum pH for growth	11	11	11	6.5–7.0	7.2	7.2	7.2–7.4
NaCl tolerance range (g l^−1^)	0–0.5	0–0.5	0–0.5	0-10	nd	nd	nd
Flagella	Polar	Polar	Polar	Polar	Polar	Polar	Polar tuft
Polyhydroxyalkanoate accumulation	+	+	+	+	+	+	−
Utilization of:							
H_2_	+	+	w	−	+	+	−
Thiosulfate	+	nt	−	−	−	+	−
Formate	w	w	w	−	−	+	+
Acetate	+	+	+	+	+	+	+
Propionate	−	nt	−	nt	−	nt	nt
Butyrate	w	w	+	nt	w	nt	nt
dl-Lactate	+	+	+	+	+	+	+
Pyruvate	+	nt	+	+	+	nt	nt
Glucose	−	+	+	+	+	+	−
Glutamate	−	nt	−	nt	+	+	nt
Glycerol	−	nt	−	−	+	+	−
Cyclohexane	+	−	w	nt	nt	nt	nt
Fumarate	+	nt	+	−	+	+	+
Electron acceptors	Oxygen thiosulfate	Oxygen nitrate	Oxygen nitrate	Oxygen nitrate	Oxygen nitrate	Oxygen	Oxygen
Fermentation by glucose	−	w	w	−	−	−	−

Antibiotic sensitivity was tested both on solid and in liquid medium for kanamycin at 50 µg ml^−1^ and for gentamycin at 10 µg ml^−1^. Growth on plates was assessed visually, while growth in liquid medium was monitored via protein concentrations using the Lowry assay [31]. All three strains were sensitive to both kanamycin and gentamycin, showing no growth on plates or in liquid medium.

Fatty acid content for strains A1^T^ and B1^T^ were analysed from acetate grown chemostat cultures. Cells were pelleted by centrifugation and stored at −20 °C. Cells for H1^T^ were grown in batch culture on acetate and harvested in the same way. Membrane lipids were extracted via a modified Bligh–Dyer protocol [32, 33] followed by saponification with 0.5 M NaOH at 70 °C for 4 h. Extracts were separated into hydrocarbon and acid fractions using solid phase extraction columns with an aminopropyl stationary phase (Supelco). Fatty acids were analysed as methyl ester derivatives. Double bond positions were determined by derivatization to dimethyl disulfide adducts following the methods of Shibamoto *et al*. [34]. Fatty acid derivatives and hydrocarbons were identified via GC-MS(Thermo Fisher Trace GC and DSQ quadrupole mass spectrometer) and quantified using a coupled flame-ionizing detector relative to an internal standard [35]. Major fatty acid constituents were consistent between strains, with strain A1^T^ containing C_16 : 0_ (3.3 %), C_16:1_ω7*c* (50.6 %), C_18 : 0_ (0.4 %) and C_18:1ω7_ (21.0 %), strain H1^T^ containing C_16 : 0_ (27.8 %), C_16:1_ω7*c* (7.1 %), C_18 : 0_ (5.9 %) and C_18:1_ω7*c* (22.6 %), and strain B1^T^ containing C_16 : 0_ (12.9 %), C_16:1_ω7*c* (34.3 %), C_18 : 0_ (3.1 %) and C_18:1_ω7*c* (20.9 %). The fatty acid profiles were determined from a single set of cultures with one replicate of each culture. The major fatty acid constituents of strains A1^T^, H1^T^ and B1^T^ (C_16 : 0_, C_16:1_ω7*c* and C_18:1_ω7*c*) are similar to those of the *Malikia granosa* P1^T^, *Hydrogenophaga flava* DSM 619^T^, and *Hydrogenophaga palleronii* DSM 63^T^, but different from those of *Macromonas bipunctata* DSM 12705^T^ (Table 3). Interestingly, an unusual monosaturated nonadecanoic acid (omega 6) was found in all strains at low levels (A1^T^ 0.9 %, H1^T^, 0.3 %, B1^T^ 1.1 %) Further, a series of saturated and monounsaturated even-chain linear hydrocarbons C_16_ to C_28_ was detected in all strains and strains A1^T^ and H1^T^ additionally contained squalene. There are some differences in the ratio of major fatty acid composition of strains A1^T^, H1^T^ and B1^T^, especially in ratio of C_16 : 0_, C_16:1_ω7*c* and C_18 : 0_. The differences enable to discriminate the strains. The respiratory quinone of strains A1^T^, H1^T^ and B1^T^ was ubiquinone, which was deduced from the coded genes of the respective genomes (kegg module M00117).

**Table 3.** Major fatty acids (%) of strains A1^T^, H1^T^, B1^T^ and their closely related type strains Strains: 1, A1^T^ (data from this study and [19]); 2, H1^T^ (data from this study and [19]); 3, B1^T^ (data from this study and [19]); 4, *Malikia granosa* P1^T^ [6]; 5, *Hydrogenophaga flava* DSM 619^T^ [11, 40]; 6, *Hydrogenophaga palleronii* DSM 63^T^ [11, 40, 41]; 7, *Macromonas bipunctata* DSM 12705^T^ [6]. For unsaturated fatty acids, the position of the double bond is located by counting from methyl (ω) end of the carbon chain. nd, Not detected.

Fatty acid	1	2	3	4	5	6	7
Saturated:							
C_14 : 0_	nd	nd	nd	1.4	2.8	0.4	2.4
C_15 : 0_	nd	nd	nd	nd	0.4	nd	1.2
C_16 : 0_	3.3	27.8	12.9	14.7	19.4	31.6	5.3
C_17 : 0_	nd	nd	nd	nd	1.3	17.8	0.8
C_18 : 0_	0.4	5.9	3.1	nd	1.5	0.6	nd
Unsaturated:							
C_15 : 1_ *ω*6*c*	nd	nd	nd	nd	nd	0.1	0.9
C_16 : 1_ *ω*5*c*	nd	nd	nd	3.2	nd*	nd	1.1
C_16 : 1_ *ω*7*c*	50.6	7.1	34.3	71.0	51.7*	25.0†	62.5
C_17 : 1_ *ω*6*c*	nd	nd	nd	nd	nd	nd	12.5
C_17 : 1_ *ω*8*c*	nd	nd	nd	nd	nd	nd	1.1
C_18 : 1_ *ω*7*c*	21.0	22.6	20.9	6.3	13.7*	19.5‡	11.9
C_19 : 1_ *ω*6*c*	0.9	0.3	1.1	nd	nd	nd	nd
Hydroxy:							
C_8 : 0_ 3-OH	nd	nd	nd	nd	1.2	3.8	0.2
C_10 : 0_ 3-OH	nd	nd	nd	nd	2.9	nd	nd
Cyclo propane:							
C_17:0_ cyc	nd	nd	nd	nd	<0.1	17.2	nd

*Summed feature comprised C_16 : 1_ or C_18 : 1_.

†Summed feature comprised C_16 : 1_ ω6*c* and/or C_16 : 1_ ω7*c*.

‡Summed feature comprised C_18 : 1_ ω6*c* and C_18 : 1_ ω7*c*.

Phase contrast images were taken using a confocal microscope (LSM8000, Zeiss) equipped with an Orca-Flach 4.0 camera (Zeiss). Scanning electron microscopy images were taken with JSM-7001 apparatus (jeol). For transmission electron microscopy, the specimens negatively strained by EM stainer (Nisshin EM) for 5 min. Cells were observed under a transmission electron microscope (JEM-ARM200F, jeol) operated at an accelerating voltage of 200 kV. These microscopic analyses revealed that the cell of strains A1^T^, H1^T^ and B1^T^ features rod-shaped, motile cells 1–3 µm long, with a single polar flagellum (Fig. 3).

**Fig. 3.**
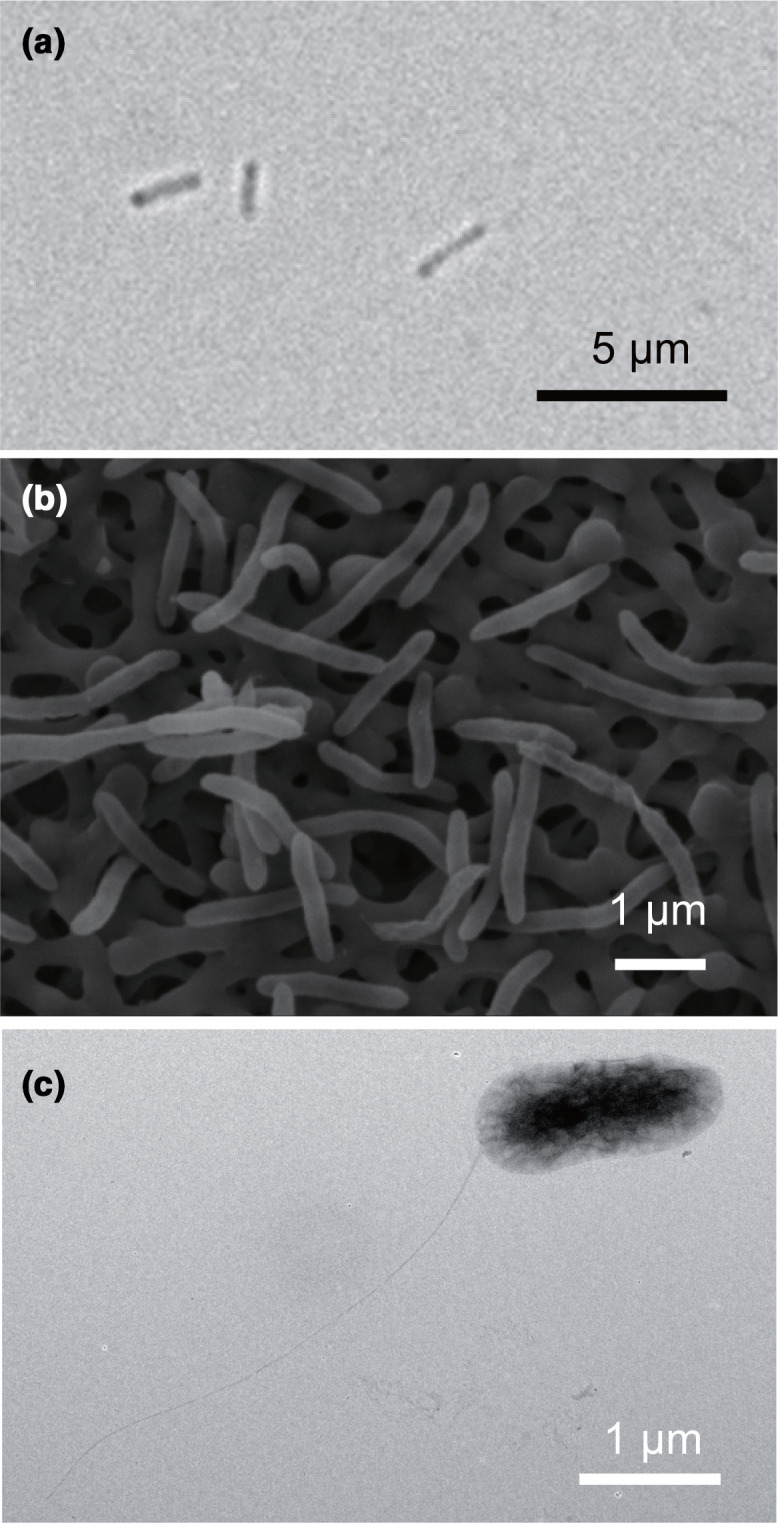
Microscopic observation of strain A1^T^. (a) Phase contrast microscopy image of strain A1^T^ grown on acetate and oxygen. The three strains are visually indistinguishable. (**b**) SEM image of strain A1^T^ on carbon filter paper. Morphologies are indistinguishable for the three strains. (**c**) TEM image of strain A1^T^ grown on acetate with oxygen.

Gram stain and catalase activity analyses revealed that all three strains were gram negative and catalase positive. The cytochromes were examined by sonicating whole cells, centrifuging at 10 000 ***g*** for 5 min, and performing a wavelength scan from 350 nm to 700 nm with a UV-Vis spectrometer (UV2600, Shimadzu). A difference spectrum was collected from air oxidized vs. dithionite reduced cell lysate. The difference spectrum showed cytochrome peaks with the maximum readings at 418–421 nm, and 550–553 nm.

Comparison of characteristics of the strains A1^T^, H1^T^ and B1^T^ with the related genera in the family *Comamonadaceae* are summarized in Table 2. While genera in the family *Comamonadaceae* harbour a remarkable phenotypic diversity, the strains A1^T^, H1^T^ and B1^T^ also share the phenotypic similarity with other genera in this family. For instance, genus *Hydrogenophaga* and strains A1^T^, H1^T^ and B1^T^ have a capability of autotrophic growth [11] and the genera *Hydrogenophaga *, *Malika* and strains A1^T^, H1^T^ and B1^T^ accumulate polyhydroxyalkanoate [6]. A notable physiological feature of strains A1^T^, H1^T^ and B1^T^ is the extremely high pH for the optimum growth (pH 11), which distinguishes it from closely related members, This high pH growth was confirmed in a continuous flow chemostat (BioFlo) at constant pH for strains A1^T^ and B1^T^; both strains showed growth at pH 11 under these conditions, and B1 continued to grow when the pH was shifted to pH 012. The optimum pH of 11 is the highest value for any prokaryote reported so far [36]. Several species in the family *Comamonadaceae* were reported as alkali tolerant or alkaliphilic, which include *Ramlibacter alkalitolerans* [37] and *Melaminivora alkalimesophila* [38] with the optimum pH 7.0 and 9.5, respectively. Genome size of strains A1^T^, H1^T^ and B1^T^ was smaller than those of the closely related strains of the family *Comamonadaceae *, while the G+C content was similar to those. Utilization of organic and inorganic substrates, as well the ability to grow on alternative electron acceptors, was variable both between the strains and in the most closely related genera. Genomic comparison of the carbon utilization genes among the three strains showed that only strain A1^T^ encodes carbon monoxide dehydrogenase, benzoyl-CoA-oxygenase and phenylacetate-CoA oxygenase, as described previously [19]. Regarding electron acceptor utilization, all three genomes encode genes for oxygen respiration, while only, H1^T^ and B1^T^ encode genes for nitrate reductase, which agrees with the experimental results (the observed ability to grow anaerobically with nitrate).

High sequence diversity of the strains A1^T^, H1^T^ and B1^T^ from members of family *Comamonadaceae* are represented in the phylogenetic dendrograms based on 16S rRNA gene sequences (Fig. 1) and concatenation of 30 conserved marker genes (Fig. 2). These relationships are also supported by the low AAI values as shown in Table 1. Thus, strains A1^T^, H1^T^ and B1^T^ merit recognition as representative of a novel genus in the family *Comamonadaceae *. Further, due to the low values of ANIb, dDDH and 16S rRNA gene identity, strains A1^T^, H1^T^ and B1^T^ each represent distinct species in this novel genus. Based on above findings, we propose that strains A1^T^, H1^T^ and B1^T^ represent three novel species in a new genus within the family *Comamonadaceae *.

## Description of *Serpentinimonas* gen. nov.

Serpentinimonas (Ser.pen.ti.ni.mo′nas. N.L. neut. n. *serpentinum* a dark green mineral produced from reaction of olivine with water; L. fem n. *monas* a shape, a monad; N.L. fem. n. *Serpentinimonas*, a monad from a serpentinizing site.).

Cells are Gram-stain-negative, rod shaped, motile cells 1–3 µm long, with a single polar flagellum. Organisms in this genus form small light-coloured (opaque creamy) colonies on plates (<1 mm). Optimum growth occurs at 30 °C at pH 11. Preferred media is without NaCl. Cells are catalase positive and sensitive to the antibiotics, kanamycin (50 µg ml^−1^) and gentamicin (10 µg ml^−1^). Fatty acid profiles are simple, containing primarily C_16 : 0_, C_16:1_ω7*c*, C_18:0,_ C_18:1_ω7*c* and C_19:1_ω6*c* fatty acids as well as linear hydrocarbons. Respiratory quinone was ubiquinone. Phylogenetically, the genus is a member of the *Comamonadaceae *. Although we proposed the novel strains as the new genus candidatus ‘Serpentinomonas’ in our previous publication, the genus name is inappropriate as Latin name, based on the publication written by H.G. Trüper [39]. Therefore, here we propose the new genus name as Serpentinimonas. The type species is *Serpentinimonas raichei*.

## Description of *Serpentinimonas raichei* sp. nov.

*Serpentinimonas raichei* (rai′che.i. N.L. gen. n. raichei, named after R. Raiche, one of the owners of The Cedars nature reserve).

In addition to the characteristics given above in the genus description, *S. raichei* has the characteristics described below. Growth occurs at 18–37 °C and pH 10.0–11.5 with optimal growth at 30 °C and pH 11.0. NaCl ranges from 0 to 0.5 g l^−1^. The DNA base composition of the type strain is 66.6 % G+C (determined from the genome). The strain grows autotrophically with hydrogen gas and calcium carbonate and heterotrophically on acetate, butyrate, lactate, pyruvate, ethanol, cyclohexane and fumarate under microaerophilic condition. The strain cannot utilize nitrate, sulphate, iron (III) hydroxide or iron (II/III) oxide as electron acceptors. The strain cannot ferment glucose. Major fatty acids are C_16:1_ω7*c* and C_18:1_ω7*c*. The respiratory quinone is ubiquinone.

The type strain, A1^T^, (=NBRC 111848^T^=DSM 103917^T^), was isolated from a highly alkaline serpentinizing spring (Barnes Spring 1) in The Cedars located in north California, USA.

## Description of *Serpentinimonas barnesii* sp. nov.

*Serpentinimonas barnesii* (bar.ne′si.i. N.L. gen. n. barnesii, named after I. Barnes, geochemist and first describer of The Cedars serpentinization site).

In addition to the characteristics given above in the genus description, the type strain has the characteristics described below. Growth occurs at 18–37 °C and pH 9.0–12.0 with optimal growth at 30 °C and pH 11.0. NaCl ranges from 0 to 0.5 g l^−1^. The DNA G+C composition of the type strain is 66.7 mol% (determined from the genome). The strain grows autotrophically with hydrogen gas and calcium carbonate and heterotrophically on acetate, butyrate, lactate, pyruvate, ethanol and fumarate under microaerophilic conditions. The strain can ferment glucose. The strain is also able to utilize glucose as an electron donor, and nitrate as an electron acceptor. Major fatty acids are C_16 : 0_ and C_18:1_ω7*c*. The respiratory quinone is ubiquinone.

The type strain, H1^T^ (=NBRC 111849^T^=DSM 103920^T^), was isolated from a highly alkaline serpentinizing spring (Barnes Spring 5) in The Cedars located in north California, USA.

## Description of *Serpentinimonas maccroryi* sp. nov.

*Serpentinimonas maccroryi* (mac.cro′ry.i. N.L. gen. n. maccroryi, named after D. McCrory, one of the owners of The Cedars nature reserve).

In addition to the characteristics given above in the genus description, the type strain has the characteristics described below. Growth occurs at 18–37 °C and pH 9.0–12.5 with optimal growth at 30 °C and pH 11.0. The strain tolerates NaCl ranges from 0 to 0.5 g l^−1^. The DNA G+C composition of the type strain is 66.7 mol% (determined from the genome). The strain grows autotrophically on formate and hydrogen gas but not on thiosulfate under microaerophilic conditions. The strain can use nitrate, but not thiosulfate as an electron acceptor. The strain can ferment glucose and grow heterotrophically on acetate, butyrate, lactate, pyruvate, ethanol, glucose and fumarate. Major fatty acids are C_16 : 0_, C_16:1_ω7*c* and C_18:1_ω7*c*. The respiratory quinone is ubiquinone.

The type strain, B1^T^ (=NBRC 111850^T^=DSM 103919^T^), was isolated from a highly alkaline serpentinizing spring (Barnes Spring 1) in The Cedars located in north California, USA.
